# *N*,*N*′-[1,3-Phenylenebis(methyl­ene)]dibenzene­sulfonamide

**DOI:** 10.1107/S1600536811040694

**Published:** 2011-10-29

**Authors:** Ejaz, Islam Ullah Khan, Hira Ahmad, William T. A. Harrison

**Affiliations:** a Materials Chemistry Laboratry, Department of Chemistry, GC University, Lahore 54000, Pakistan; b Department of Chemistry, University of Aberdeen, Meston Walk, Aberdeen AB24 3UE, Scotland

## Abstract

The complete mol­ecule of the title compound, C_20_H_20_N_2_O_4_S_2_, is generated by crystallographic twofold symmetry, with two C atoms lying on the rotation axis. The dihedral angle between the central benzene ring and the pendant ring is 68.42 (6)° and the dihedral angle between the pendant rings is 45.11 (5)°. The torsion angles for the C—S—N—C and S—N—C—C fragments are −73.22 (15) and −150.45 (13)°, respectively. In the crystal, mol­ecules are linked by N—H⋯O hydrogen bonds, generating corrugated (001) sheets. Aromatic π–π stacking [centroid–centroid separation = 3.8925 (12) and 3.9777 (12) Å] and weak C—H⋯O inter­actions also occur.

## Related literature

For a related structure, see: Khan *et al.* (2011 ▶).
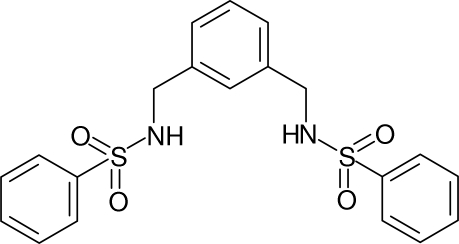


## Experimental

### 

#### Crystal data

C_20_H_20_N_2_O_4_S_2_
*M* *_r_* = 416.50
Monoclinic, 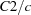

*a* = 16.4319 (6) Å
*b* = 7.8024 (3) Å
*c* = 16.1217 (6) Å
β = 111.543 (2)°
*V* = 1922.54 (12) Å^3^
*Z* = 4
Mo *K*α radiation
μ = 0.31 mm^−1^
*T* = 296 K
0.15 × 0.11 × 0.08 mm

#### Data collection

Bruker APEXII CCD diffractometer
8653 measured reflections
2346 independent reflections
1841 reflections with *I* > 2σ(*I*)
*R*_int_ = 0.024

#### Refinement

*R*[*F* ^2^ > 2σ(*F* ^2^)] = 0.037
*wR*(*F* ^2^) = 0.103
*S* = 1.07
2346 reflections
131 parameters
H atoms treated by a mixture of independent and constrained refinement
Δρ_max_ = 0.29 e Å^−3^
Δρ_min_ = −0.34 e Å^−3^

### 

Data collection: *APEX2* (Bruker, 2007 ▶); cell refinement: *SAINT* (Bruker, 2007 ▶); data reduction: *SAINT*; program(s) used to solve structure: *SHELXS97* (Sheldrick, 2008 ▶); program(s) used to refine structure: *SHELXL97* (Sheldrick, 2008 ▶); molecular graphics: *ORTEP-3* (Farrugia, 1997 ▶); software used to prepare material for publication: *SHELXL97*.

## Supplementary Material

Crystal structure: contains datablock(s) I, global. DOI: 10.1107/S1600536811040694/su2323sup1.cif

Structure factors: contains datablock(s) I. DOI: 10.1107/S1600536811040694/su2323Isup2.hkl

Supplementary material file. DOI: 10.1107/S1600536811040694/su2323Isup3.cml

Additional supplementary materials:  crystallographic information; 3D view; checkCIF report

## Figures and Tables

**Table 1:** Hydrogen-bond geometry (Å, °)

*D*—H⋯*A*	*D*—H	H⋯*A*	*D*⋯*A*	*D*—H⋯*A*
N1—H1⋯O2^i^	0.81 (2)	2.16 (2)	2.960 (2)	166 (2)
C5—H5⋯O1^ii^	0.93	2.55	3.461 (2)	166
C10—H10⋯O1^iii^	0.93	2.57	3.379 (3)	146

Symmetry codes: (i) 
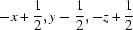
; (ii) 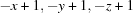; (iii) 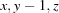.

**Fig. 1.**
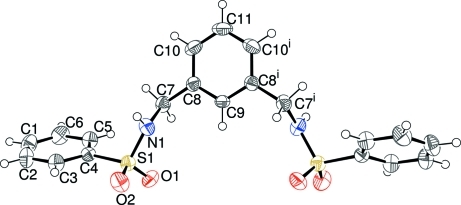
The molecular structure of the title molecule, showing the numbering cheme and 50% displacement ellipsoids [Symmetry code: (i) 1–x, y, 1/2 - z].

## supplementary crystallographic information

### Comment

As part of our ongoing structural studies of sulfonamides (Khan *et al.*, 2011), the synthesis and structure of the title compound are described herein.

The complete molecule of the title compound is generated by crystallographic twofold symmetry (Fig. 1), with atoms C9 and C11 lying on the rotation axis. The diehdral angle between the central benzene ring and the pendant ring is 68.42 (6)°. The dihedral angle between the two pendant rings is 45.11 (5)°. The conformation of the C4—S1—N1—C7 fragment is approximately *gauche* [-73.22 (15)°] whereas the torsion angle for S1—N1—C8—C9 of -150.45 (13)° indicates a conformation between *gauche* and *anti*. The bond-angle sum for N1 of 350.6° seems to indicate a valence state close to *sp*^2^ hybridization.

In the crystal, the molecules are linked by N—H···O hydrogen bonds (Table 1), to generate corrugated (010) sheets (Fig. 2). Weak aromatic π-π stacking [centroid-centroid separation = 3.8925 (12) and 3.9777 (12) Å] occurs between the layers and weak C—H···O interactions may help to consolidate the packing.

### Experimental

The *m*-xylylenediamine (0.132 ml, 1.0 mmol) was mixed with 20 ml distilled water in a 50 ml round bottom flask. Benzene sulfonyl chloride (0.255 ml, 2.0 mmol) was added while maintaining the pH of the reaction mixture at ca. 9.0 using sodium carbonate solution (3%) and the mixture was stirred for five hours. The white precipitated product was filtered, washed, dried and crystallized from methanol to generate colourless blocks of the title compound.

### Refinement

The N-bound H atom was located in a difference map and its position was freely refined with the constraint *U*_iso_(H) = 1.2*U*_eq_(N). The C-bound hydrogen atoms were placed in calculated positions (C—H = 0.93–0.97 Å) and refined as riding atoms with *U*_iso_(H) = 1.2*U*_eq_(C).

### Crystal data

**Table:**

C_20_H_20_N_2_O_4_S_2_	*F*(000) = 872
*M**_r_* = 416.50	*D*_x_ = 1.439 Mg m^−^^3^
Monoclinic, *C*2/*c*	Mo *K*α radiation, λ = 0.71073 Å
Hall symbol: -C 2yc	Cell parameters from 2346 reflections
*a* = 16.4319 (6) Å	θ = 2.9–28.3°
*b* = 7.8024 (3) Å	µ = 0.31 mm^−^^1^
*c* = 16.1217 (6) Å	*T* = 296 K
β = 111.543 (2)°	Block, colourless
*V* = 1922.54 (12) Å^3^	0.15 × 0.11 × 0.08 mm
*Z* = 4	

### Data collection

**Table:**

Bruker APEXII CCD diffractometer	1841 reflections with *I* > 2σ(*I*)
Radiation source: fine-focus sealed tube	*R*_int_ = 0.024
graphite	θ_max_ = 28.3°, θ_min_ = 2.9°
ω scans	*h* = −21→21
8653 measured reflections	*k* = −9→10
2346 independent reflections	*l* = −21→21

### Refinement

**Table:**

Refinement on *F*^2^	Primary atom site location: structure-invariant direct methods
Least-squares matrix: full	Secondary atom site location: difference Fourier map
*R*[*F*^2^ > 2σ(*F*^2^)] = 0.037	Hydrogen site location: inferred from neighbouring sites
*wR*(*F*^2^) = 0.103	H atoms treated by a mixture of independent and constrained refinement
*S* = 1.07	*w* = 1/[σ^2^(*F*_o_^2^) + (0.0466*P*)^2^ + 1.1784*P*] where *P* = (*F*_o_^2^ + 2*F*_c_^2^)/3
2346 reflections	(Δ/σ)_max_ = 0.001
131 parameters	Δρ_max_ = 0.29 e Å^−^^3^
0 restraints	Δρ_min_ = −0.34 e Å^−^^3^

### Special details

**Table:**

Geometry. All e.s.d.'s (except the e.s.d. in the dihedral angle between two l.s. planes) are estimated using the full covariance matrix. The cell e.s.d.'s are taken into account individually in the estimation of e.s.d.'s in distances, angles and torsion angles; correlations between e.s.d.'s in cell parameters are only used when they are defined by crystal symmetry. An approximate (isotropic) treatment of cell e.s.d.'s is used for estimating e.s.d.'s involving l.s. planes.
Refinement. Refinement of *F*^2^ against ALL reflections. The weighted *R*-factor *wR* and goodness of fit *S* are based on *F*^2^, conventional *R*-factors *R* are based on *F*, with *F* set to zero for negative *F*^2^. The threshold expression of *F*^2^ > σ(*F*^2^) is used only for calculating *R*-factors(gt) *etc*. and is not relevant to the choice of reflections for refinement. *R*-factors based on *F*^2^ are statistically about twice as large as those based on *F*, and *R*- factors based on ALL data will be even larger.

### Fractional atomic coordinates and isotropic or equivalent isotropic displacement parameters (Å^2^)

**Table:**

	*x*	*y*	*z*	*U*_iso_*/*U*_eq_	
C1	0.23478 (13)	0.4749 (3)	0.57485 (13)	0.0492 (5)	
H1A	0.2133	0.4616	0.6204	0.059*	
C2	0.18163 (12)	0.5397 (3)	0.49388 (14)	0.0493 (5)	
H2	0.1239	0.5677	0.4846	0.059*	
C3	0.21285 (11)	0.5637 (3)	0.42623 (12)	0.0405 (4)	
H3	0.1766	0.6069	0.3713	0.049*	
C4	0.29908 (10)	0.5225 (2)	0.44139 (10)	0.0313 (4)	
C5	0.35276 (12)	0.4531 (2)	0.52240 (11)	0.0396 (4)	
H5	0.4102	0.4230	0.5318	0.048*	
C6	0.31953 (13)	0.4296 (3)	0.58870 (12)	0.0481 (5)	
H6	0.3548	0.3828	0.6431	0.058*	
C7	0.43547 (11)	0.2757 (2)	0.36899 (11)	0.0378 (4)	
H7A	0.4162	0.1957	0.4041	0.045*	
H7B	0.4827	0.3439	0.4096	0.045*	
C8	0.46828 (10)	0.1780 (2)	0.30692 (11)	0.0332 (4)	
C9	0.5000	0.2653 (3)	0.2500	0.0320 (5)	
H9	0.5000	0.3845	0.2500	0.038*	
C10	0.46821 (12)	0.0009 (3)	0.30605 (13)	0.0429 (4)	
H10	0.4467	−0.0593	0.3434	0.052*	
C11	0.5000	−0.0871 (4)	0.2500	0.0504 (7)	
H11	0.5000	−0.2063	0.2500	0.061*	
S1	0.34361 (3)	0.56500 (6)	0.35953 (3)	0.03403 (15)	
N1	0.36255 (9)	0.3882 (2)	0.31843 (10)	0.0382 (4)	
H1	0.3187 (13)	0.339 (3)	0.2865 (14)	0.046*	
O1	0.42667 (8)	0.64544 (18)	0.40359 (9)	0.0459 (3)	
O2	0.27803 (9)	0.65172 (19)	0.28749 (8)	0.0502 (4)	

### Atomic displacement parameters (Å^2^)

**Table:**

	*U*^11^	*U*^22^	*U*^33^	*U*^12^	*U*^13^	*U*^23^
C1	0.0510 (11)	0.0624 (14)	0.0390 (10)	−0.0120 (10)	0.0223 (9)	−0.0015 (9)
C2	0.0371 (10)	0.0647 (14)	0.0507 (11)	−0.0021 (9)	0.0213 (9)	−0.0029 (10)
C3	0.0355 (9)	0.0490 (12)	0.0342 (9)	0.0017 (8)	0.0097 (7)	0.0017 (8)
C4	0.0330 (8)	0.0318 (9)	0.0282 (7)	−0.0021 (6)	0.0101 (6)	−0.0021 (6)
C5	0.0362 (9)	0.0459 (11)	0.0343 (8)	0.0004 (7)	0.0101 (7)	0.0019 (7)
C6	0.0526 (11)	0.0575 (13)	0.0307 (9)	−0.0038 (9)	0.0110 (8)	0.0074 (8)
C7	0.0364 (9)	0.0416 (11)	0.0367 (9)	0.0013 (7)	0.0151 (7)	0.0044 (8)
C8	0.0264 (7)	0.0335 (10)	0.0379 (8)	−0.0002 (6)	0.0098 (7)	0.0026 (7)
C9	0.0316 (11)	0.0233 (12)	0.0409 (12)	0.000	0.0131 (10)	0.000
C10	0.0406 (9)	0.0337 (10)	0.0530 (11)	−0.0052 (8)	0.0155 (9)	0.0091 (9)
C11	0.0536 (16)	0.0234 (14)	0.0696 (19)	0.000	0.0171 (15)	0.000
S1	0.0348 (2)	0.0367 (3)	0.0307 (2)	0.00170 (17)	0.01223 (17)	0.00359 (17)
N1	0.0313 (7)	0.0452 (10)	0.0358 (8)	0.0011 (6)	0.0095 (6)	−0.0079 (7)
O1	0.0439 (7)	0.0438 (8)	0.0516 (7)	−0.0117 (6)	0.0195 (6)	0.0002 (6)
O2	0.0544 (8)	0.0605 (10)	0.0357 (7)	0.0195 (7)	0.0165 (6)	0.0149 (6)

### Geometric parameters (Å, °)

**Table:**

C1—C2	1.373 (3)	C7—H7A	0.9700
C1—C6	1.373 (3)	C7—H7B	0.9700
C1—H1A	0.9300	C8—C10	1.382 (3)
C2—C3	1.378 (3)	C8—C9	1.389 (2)
C2—H2	0.9300	C9—C8^i^	1.389 (2)
C3—C4	1.385 (2)	C9—H9	0.9300
C3—H3	0.9300	C10—C11	1.381 (2)
C4—C5	1.390 (2)	C10—H10	0.9300
C4—S1	1.7596 (17)	C11—C10^i^	1.381 (2)
C5—C6	1.379 (3)	C11—H11	0.9300
C5—H5	0.9300	S1—O2	1.4314 (12)
C6—H6	0.9300	S1—O1	1.4315 (13)
C7—N1	1.467 (2)	S1—N1	1.6092 (16)
C7—C8	1.506 (2)	N1—H1	0.81 (2)
			
C2—C1—C6	120.16 (18)	H7A—C7—H7B	108.1
C2—C1—H1A	119.9	C10—C8—C9	118.86 (17)
C6—C1—H1A	119.9	C10—C8—C7	120.91 (16)
C1—C2—C3	120.73 (18)	C9—C8—C7	120.22 (17)
C1—C2—H2	119.6	C8^i^—C9—C8	121.3 (2)
C3—C2—H2	119.6	C8^i^—C9—H9	119.4
C2—C3—C4	118.88 (16)	C8—C9—H9	119.4
C2—C3—H3	120.6	C11—C10—C8	120.31 (19)
C4—C3—H3	120.6	C11—C10—H10	119.8
C3—C4—C5	120.78 (16)	C8—C10—H10	119.8
C3—C4—S1	120.29 (13)	C10—C11—C10^i^	120.4 (3)
C5—C4—S1	118.89 (13)	C10—C11—H11	119.8
C6—C5—C4	119.00 (17)	C10^i^—C11—H11	119.8
C6—C5—H5	120.5	O2—S1—O1	119.47 (9)
C4—C5—H5	120.5	O2—S1—N1	105.84 (8)
C1—C6—C5	120.41 (17)	O1—S1—N1	106.67 (8)
C1—C6—H6	119.8	O2—S1—C4	107.53 (8)
C5—C6—H6	119.8	O1—S1—C4	107.08 (8)
N1—C7—C8	110.62 (13)	N1—S1—C4	110.10 (8)
N1—C7—H7A	109.5	C7—N1—S1	121.73 (12)
C8—C7—H7A	109.5	C7—N1—H1	114.8 (15)
N1—C7—H7B	109.5	S1—N1—H1	114.1 (15)
C8—C7—H7B	109.5		
			
C6—C1—C2—C3	−1.4 (3)	C7—C8—C10—C11	−178.78 (13)
C1—C2—C3—C4	−0.4 (3)	C8—C10—C11—C10^i^	−0.31 (11)
C2—C3—C4—C5	1.9 (3)	C3—C4—S1—O2	3.65 (17)
C2—C3—C4—S1	−175.61 (15)	C5—C4—S1—O2	−173.94 (14)
C3—C4—C5—C6	−1.6 (3)	C3—C4—S1—O1	133.19 (15)
S1—C4—C5—C6	175.99 (15)	C5—C4—S1—O1	−44.39 (17)
C2—C1—C6—C5	1.8 (3)	C3—C4—S1—N1	−111.21 (15)
C4—C5—C6—C1	−0.3 (3)	C5—C4—S1—N1	71.20 (16)
N1—C7—C8—C10	−121.05 (17)	C8—C7—N1—S1	−150.45 (13)
N1—C7—C8—C9	59.57 (18)	O2—S1—N1—C7	170.85 (14)
C10—C8—C9—C8^i^	−0.30 (11)	O1—S1—N1—C7	42.63 (16)
C7—C8—C9—C8^i^	179.09 (15)	C4—S1—N1—C7	−73.22 (15)
C9—C8—C10—C11	0.6 (2)		

Symmetry codes: (i) −*x*+1, *y*, −*z*+1/2.

### Hydrogen-bond geometry (Å, °)

**Table:**

*D*—H···*A*	*D*—H	H···*A*	*D*···*A*	*D*—H···*A*
N1—H1···O2^ii^	0.81 (2)	2.16 (2)	2.960 (2)	166 (2)
C5—H5···O1^iii^	0.93	2.55	3.461 (2)	166
C10—H10···O1^iv^	0.93	2.57	3.379 (3)	146

Symmetry codes: (ii) −*x*+1/2, *y*−1/2, −*z*+1/2; (iii) −*x*+1, −*y*+1, −*z*+1; (iv) *x*, *y*−1, *z*.
